# Novel Low-Density Lipoprotein Cholesterol Reduction Therapies for the Secondary Prevention of Cardiovascular Disease

**DOI:** 10.31083/j.rcm2410286

**Published:** 2023-10-08

**Authors:** Xing Wang, Dingke Wen, Mei Fang, Chao You, Lu Ma

**Affiliations:** ^1^Department of Neurosurgery, West China Hospital, Sichuan University, 610041 Chengdu, Sichuan, China; ^2^West China Medicine School, Sichuan University, 610041 Chengdu, Sichuan, China

**Keywords:** monoclonal antibody, low-density lipoprotein, proprotein convertase subtilisin/kexin type 9, secondary prevention, bempedoic acid

## Abstract

**Background:**

To date, optimal agents for low-density lipoprotein cholesterol (LDL-C) reduction in patients with established atherosclerotic cardiovascular disease are still being explored. Thus, we evaluated the efficiency of novel LDL-C-lowering therapies in the secondary prevention of cardiovascular events.

**Methods:**

We included randomized clinical trials (RCTs) that explored the effects of different LDL-C lowering agents including alirocumab, evolocumab, and bempedoic acid in adult patients with cardiovascular disease. Several databases were searched from inception through 2022. The safety endpoint includes new-onset diabetes, serious adverse events, and neurocognitive disorders with at least 1 year of follow-up. The efficacy outcomes included composite adverse cardiovascular outcomes, all-cause death, and cardiovascular death.

**Results:**

Seven RCTs comprising 53,106 patients were included in this research. Bempedoic acid ranked first in reducing the risk of new-onset diabetes (risk ratio [RR] 0.72, 95% credible interval [CrI] 0.52–0.99) and risk of the composite cardiovascular outcome (RR 0.75, 95% CrI 0.57–0.99). Meta-regression analysis demonstrated that elevated risk of new-onset diabetes was positively correlated with a significant reduction in LDL-C levels (*p = *0.03). All treatment agents were associated with a decreased risk of a composite adverse cardiovascular outcome.

**Conclusions:**

The present analysis showed that bempedoic acid ranked first in reducing the risk of a composite cardiovascular outcome. In addition, it ranked first in reducing the risk of new-onset diabetes compared with placebo and evolocumab. Our analysis also suggests that the increased risk of new-onset diabetes might be associated with a reduction in LDL-C levels. Besides, the present analysis found that alirocumab ranked first in decreasing all-cause mortality and cardiovascular mortality.

## 1. Introduction

Secondary prevention of cardiovascular disease is a highly critical problem. Studies have reported that patients with existing cardiovascular disease have a higher risk of recurrent cardiovascular events, resulting in an elevated incidence of mortality [1, 2]. A previous cohort study reported that the longer-term risk of recurrent cardiovascular events is about 46%. In addition, diabetes mellitus is associated with recurrence [3].

Statins have been the preferred choice to lower cholesterol levels for decades. Unfortunately, a growing number of patients do not meet the recommended low-density lipoprotein cholesterol (LDL-C) goals or are incapable of tolerating this agent [4, 5]. Recently, non-statin cholesterol-lowering agents (e.g., proprotein convertase subtilisin/kexin type 9 [PCSK9] monoclonal antibodies, bempedoic acid) have shown cardiovascular benefits [6, 7]. These findings provide a positive message for patients who are unable to tolerate statins or are unable to control LDL-C levels at the maximally tolerated statin dose. However, the comparative effects of different novel agents in reducing LDL-C in patients with the atherosclerotic cardiovascular disease remain unknown.

The PCSK9 monoclonal antibody is more effective than bempedoic acid in lowering LDL-C; however, this does not mean that patients treated with PCSK9 monoclonal antibody have a better clinical outcome than those treated with bempedoic acid [8]. For example, recent studies have observed an association between circulating PCSK9 concentration and the progression of new-onset diabetes [9, 10, 11]. While bempedoic acid can decrease the incidence of new-onset diabetes [12, 13], it has been difficult to determine whether reduced LDL-C levels are associated with an increased risk of developing new-onset diabetes with this treatment.

In light of the above issues, we designed a network meta-analysis with meta-regression to evaluate the efficacy of novel LDL-C-lowering therapies on the secondary prevention of cardiovascular events and new-onset diabetes. Our results may be useful for clinicians in their daily practice as well as in the development of optimal clinical guidelines.

## 2. Methods

### 2.1 Eligibility Criteria

Eligible studies complied with the following standards for patients, interventions, comparisons, outcomes, and study design. All or a subset of adult patients (age ≥18 years) had coronary heart disease or risk equivalent of the disease. Patients were treated with maximally tolerated or moderate-to-high intensity statin. The interventions were PCSK9 monoclonal antibodies (including alirocumab and evolocumab) and bempedoic acid with a placebo as a comparison. The safety endpoint included new-onset diabetes, serious adverse events (SAEs), and neurocognitive disorders with at least 1 year of follow-up. The efficacy outcomes included composite adverse cardiovascular outcomes, all-cause death, and cardiovascular death. The duration of follow-up was at least 1 year or 48 weeks. The study design was randomized clinical trials (RCTs).

### 2.2 Search Strategy and Study Identification

A thorough literature search was performed using the Cochrane CENTRAL, Embase, and Medline databases from inception through July 15, 2022. In addition, a thorough search of the references to the included studies and relevant reviews in the same field was also conducted to find any available trials. In addition, the WHO Clinical Trials Registry Platform and the US National Library of Medicine Clinical Trials Registry Platform were applied to identify ongoing studies. The details of the search strategy can be found in **Supplementary Table 1**.

Briefly, two reviewers selected studies and extracted data on the study characteristics from eligible trials using a form as previously described [14]. In the event of any discrepancy, the issue was resolved by thorough discussion among the study team. We contacted corresponding author of the study to request more information if there was unclear information.

### 2.3 Quality Assessment 

The risk of bias for all trials was evaluated by the Cochrane Collaboration Risk of Bias tool according to seven domains [15]. The Grading of Recommendations Assessment, Development, and Evaluation working group (GRADE) tool was applied to judge the quality of evidence for the preferred outcomes [16]. A total of five aspects including inconsistency, imprecision, publication bias, global risk of bias, and indirectness were assessed.

### 2.4 Data Synthesis 

The present analyses contained a three-node analysis (PCSK9 monoclonal antibodies vs. bempedoic acid vs. placebo) and a four-node analysis (alirocumab vs. evolocumab vs. bempedoic acid vs. placebo). To integrate indirect evidence, Bayesian network meta-analyses with a consistency model were conducted using R software (version 4.1.1, R Core Team, Boston, MA, USA). The models were initially built on the basis of 30,000 iterations after removing the first 10,000. We also modified the parameters until a satisfactory convergence was achieved. Moreover, we conducted meta-regression analyses to explore the association between baseline LDL-C level, the difference in LDL-C change, and the incidence of the concerned outcomes. In the network meta-analysis, rank plots were performed to estimate the intervention hierarchy. The heterogeneity of each model was evaluated by the $I^{2}$ test. A global $I^{2}$ value of more than 50% was considered substantial. A two-sided *p*-value < 0.05 was considered statistically significant.

This study was registered in the PROSPERO database (CRD42022340569) and the Open Science Framework portal (https://osf.io/fdsbr). The methods and reporting of the systematic review and meta-analysis followed the Preferred Reporting Items for Systematic Reviews and Meta-Analyses for Network Meta-Analyses Extension Statement [17]. All statistical analyses were carried out with relevant packages in R software (version 4.1.1, R Core Team, Boston, MA, USA) and Review Manager (version 5.4.0, the Cochrane Collaboration, The Nordic Cochrane Centre, Copenhagen, Denmark).

## 3. Results

### 3.1 Study Selection and Study Characteristics

The systematic electronic literature search yielded 1632 publications (**Supplementary Fig. 1**). After the exclusion of studies according to pre-specified criteria, seven trials including 53,106 patients were included in the analyses [18, 19, 20, 21, 22, 23, 24].

Details of the included RCTs are presented in Table 1. In brief, these trials were published from 2015 to 2022. The number of participants in each trial ranged from 300 to 27,564 patients. The age of patients in the included studies ranged from 58.6 to 66.1 years old. Three trials compared alirocumab versus placebo; two trials compared bempedoic acid versus placebo; and two trials compared evolocumab versus placebo.

**Table 1.** **Characteristics of studies included in the systematic review**.

Trial	Patients, n	Male*	Age, years*	BMI, ${\mathrm{kg/m}}^{2}$*	Baseline LDL-C mg/dL*	CHD/ASCVD	HeFH	Diabetes	Statin use	High dose statins	Intervention	Follow up, weeks
ODYSSEY LONG TERM 2015 [24]	2341	63.3%	60.6	30.5	121.9	68.6/82.4%	17.7%	34.6%	>99.9%	46.8%	Alirocumab 150 mg every 2 weeks	78 weeks
GLAGOV 2016 [23]	968	72.2%	59.8	29.5	92.4	100/100%	NA	20.9%	100%	58.9%	Evolocumab 420 mg every month	78 weeks
FOURIER 2017 [22]	27,564	75.4%	62.5	NR	92	81.1/100%	NA	36.6%	100%	69.3%	Evolocumab 140 mg every 2 weeks or 420 mg every month	2.2 years
ODYSSEY OUTCOMES 2018 [21]	18,924	74.8%	58.6	28.5	92	100/100%	NA	28.8%	100%	NR	Alirocumab 75 mg every 2 weeks	2.8 years
PACMAN-AMI 2022 [18]	300	81.0%	58.6	28.2	150.9	100/100%	NA	10.3%	100%	97.4%	Alirocumab 150 mg every 2 weeks	52 weeks
CLEAR Harmony 2019 [19]	2230	63.0%	66.1	NR	103.2	NR/97.6%	3.5%	28.6%	100%	49.9%	Bempedoic acid 180 mg daily	52 weeks
CLEAR Wisdom 2019 [20]	779	63.7%	64.3	30.6	120.4	81.8/94.5%	5.5%	30.3%	100%	53.0%	Bempedoic acid 180 mg daily	52 weeks

NR, not reported; NA, not applicable; BMI, body mass index; LDL-C, low-density lipoprotein cholesterol; CHD, coronary heart disease; ASCVD, atherosclerotic cardiovascular disease; HeFH, heterozygous familial hypercholesterolemia. *from the control group.

### 3.2 Safety Outcome

**Fig. 1.**
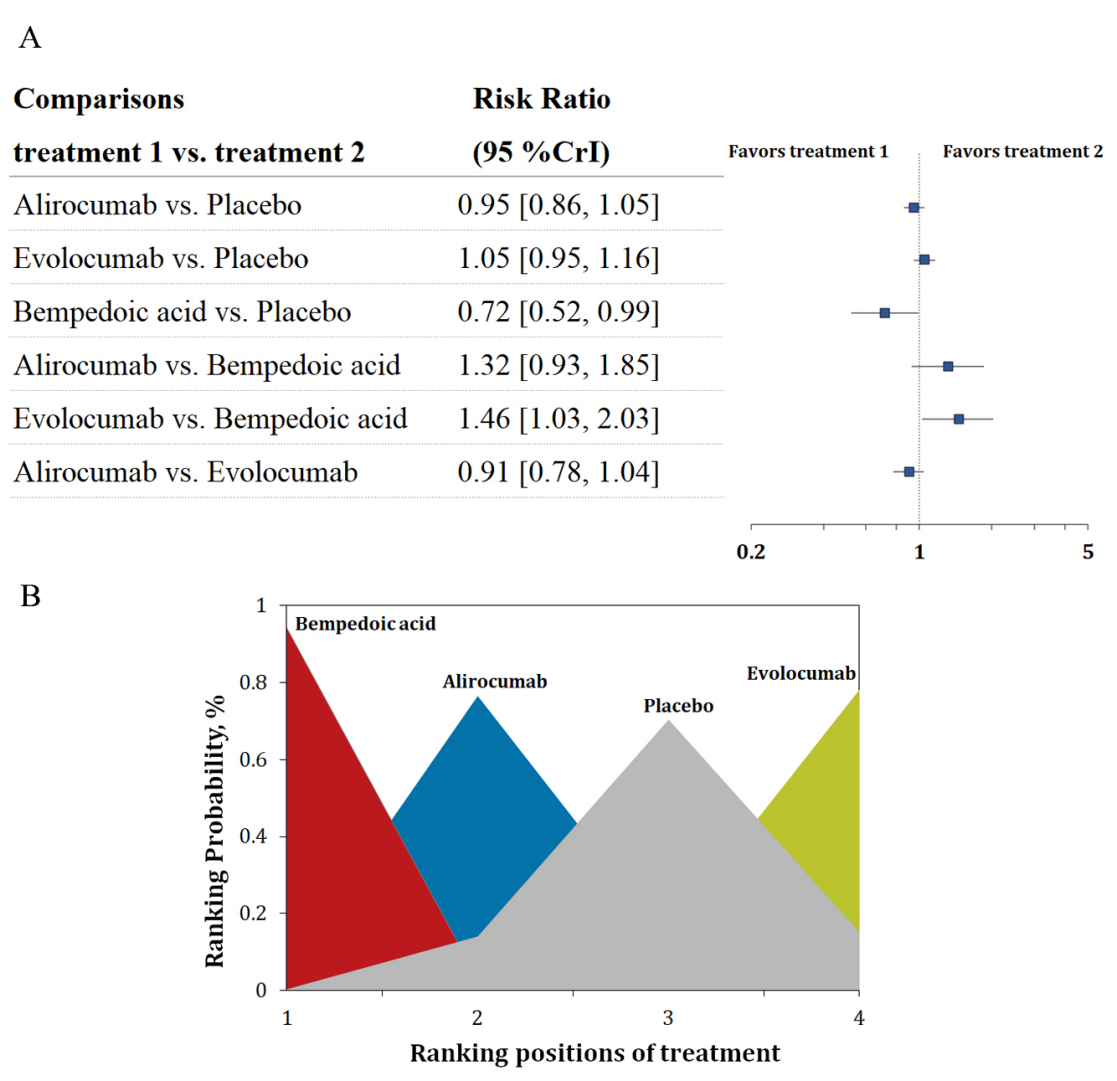
**Overview of the new-onset diabetes outcome.** (A) Summary RRs with 95% CrIs for new-onset diabetes. (B) Ranking plot showing the SUCRA values of each treatment agent: 97% for bempedoic acid, 61% for alirocumab, and 9% for evolocumab. SUCRA, surface under the cumulative ranking curve; RRs, risk ratios; CrIs, credible intervals.

**Fig. 2.**
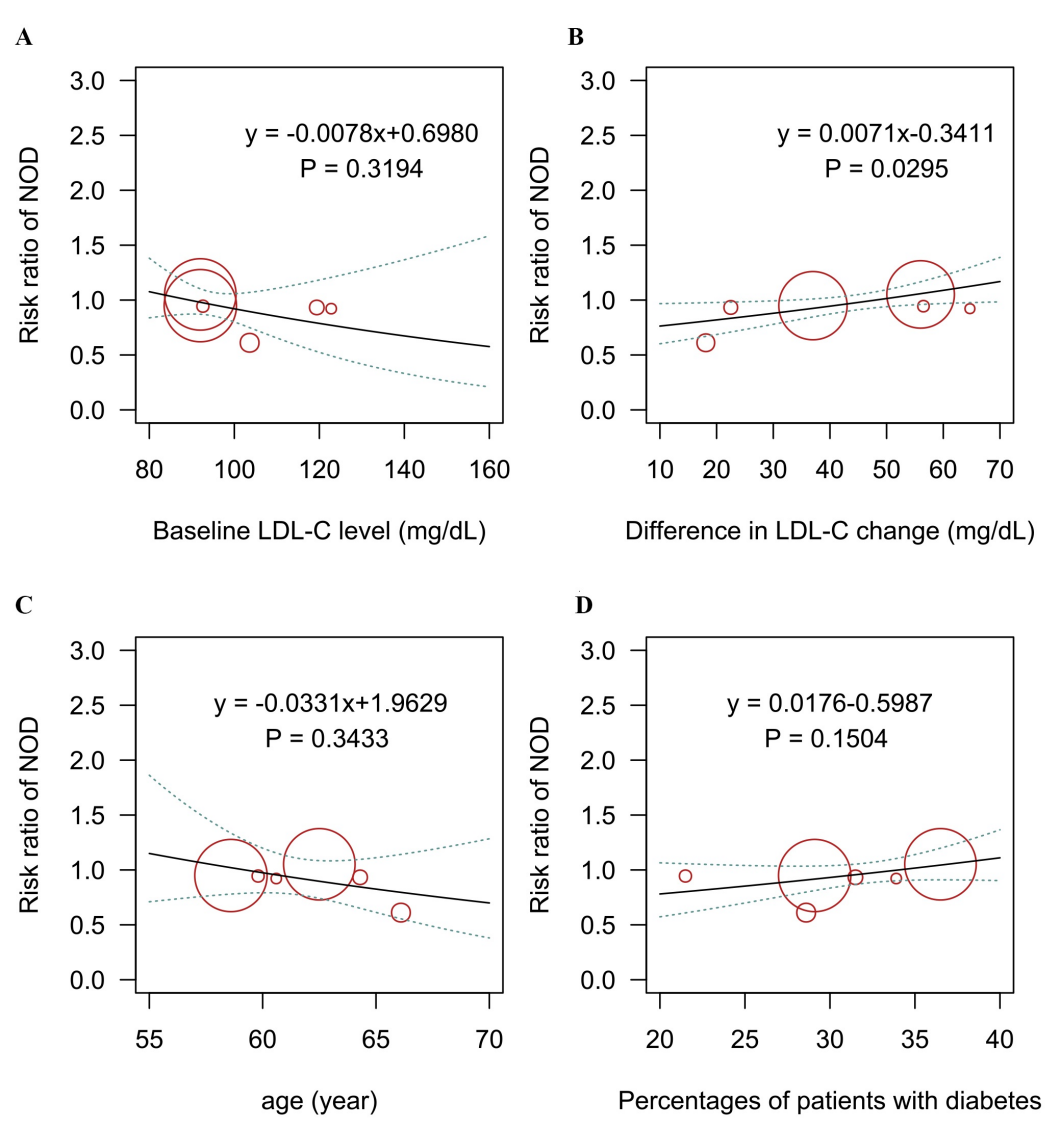
**Meta-regression analysis for the association of (A) baseline LDL-C level, (B) difference in LDL-C change, (C) age, (D) percentage of patients with diabetes on the risk of new-onset diabetes outcome.** LDL-C, low-density lipoprotein cholesterol; NOD, new-onset diabetes.

Six trials with 35,614 patients reported data on new-onset diabetes. Three-node analysis revealed bempedoic acid was preferable to PCSK9 monoclonal antibodies in decreasing new-onset diabetes (relative risk [RR] 1.38, 95% credible interval [CrI] 1.00–1.92; Table 2). Four-node analysis showed that bempedoic acid was the best agent for reducing the incidence of new-onset diabetes (Fig. 1A,B; **Supplementary Table 2**) with statistical significance (RR 0.72, 95% CrI 0.52–0.99, surface under the cumulative ranking curve [SUCRA] 0.97). The analysis also revealed that bempedoic acid was superior to evolocumab in decreasing the risk of new-onset diabetes (RR 0.68, 95% CrI 0.49–0.97). Meta-regression analyses were performed to examine the relationship between baseline LDL-C level, the difference in LDL-C change, and the incidence of new-onset diabetes. The outcomes showed a statistically significant association between increased risks and differences in LDL-C change (*p* = 0.03; Fig. 2).

**Table 2.** **Aggregated RR and relative CrI of different treatment options in patients with cardiovascular disease derived from a three-node network meta-analysis**.

Intervention		RR (95% CrI) estimates derived from NMA			SUCRA		
		PCSK9 monoclonal antibodies vs. placebo	Bempedoic acid vs. placebo	PCSK9 monoclonal antibodies vs. bempedoic acid	PCSK9 monoclonal antibodies	Bempedoic acid	Placebo
Safety outcomes							
	New-onset diabetes	1.00 (0.93, 1.07)	0.72 (0.53, 0.99)	1.38 (1.00, 1.92)	0.28	0.97	0.24
	Serious adverse events	0.97 (0.94, 1.01)	1.06 (0.89, 1.27)	0.92 (0.77, 1.10)	0.88	0.22	0.40
	Neurocognitive disorders	0.99 (0.87, 1.13)	0.93 (0.40, 2.30)	1.07 (0.43, 2.49)	0.49	0.57	0.44
Efficacy outcomes							
	Composite cardiovascular outcome	0.85 (0.81, 0.90)	0.75 (0.57, 0.99)	1.14 (0.85, 1.51)	0.60	0.89	0.01
	All-cause death	0.94 (0.86, 1.04)	2.53 (0.94, 8.98)	0.37 (0.10, 1.01)	0.93	0.03	0.54
	Cardiovascular death	0.95 (0.84, 1.07)	1.78 (0.52, 8.64)	0.53 (0.11, 1.83)	0.82	0.18	0.50

NMA, network meta-analysis; CrI, credible interval; SUCRA, surface under the cumulative ranking curve; RR, relative risk; PCSK9, proprotein convertase subtilisin/kexintype 9.

**Fig. 3.**
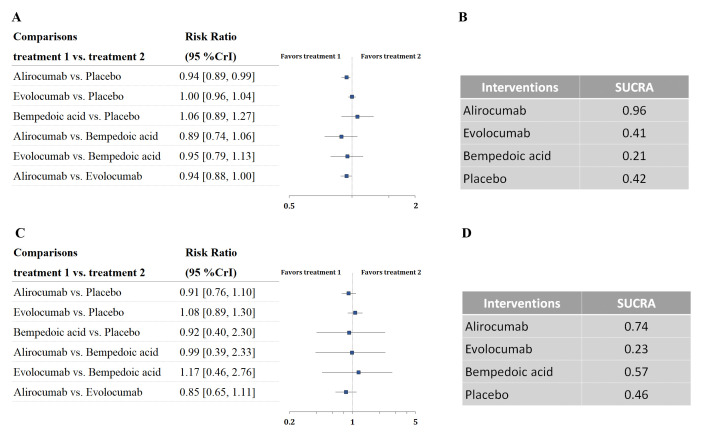
**Overview of other safety outcomes.** (A,B) Summary RRs with 95% CrIs and SUCRA values for SAE. (C,D) Summary RRs with 95% CrIs and SUCRA values for neurocognitive disorders. CrI, credible interval; SAE, serious adverse events; SUCRA, surface under the cumulative ranking curve; RRs, risk ratios.

Five trials with 51,765 patients reported data on the SAEs. The results are presented in Fig. 3A,B. Alirocumab was regarded as the first agent to decrease all-cause mortality, with statistical significance (RR 0.94, 95% CrI 0.89–0.99, SUCRA 0.96). The analysis also revealed that alirocumab was superior to evolocumab in decreasing the incidence of SAE (RR 0.94, 95% CrI 0.88–1.00).

Six trials with 52,733 patients reported data on neurocognitive disorders (Fig. 3C,D). The analysis did not reveal any statistical significance in any of the comparisons. Alirocumab was found to be the first treatment regimen in this outcome.

### 3.3 Efficacy Outcomes 

**Fig. 4.**
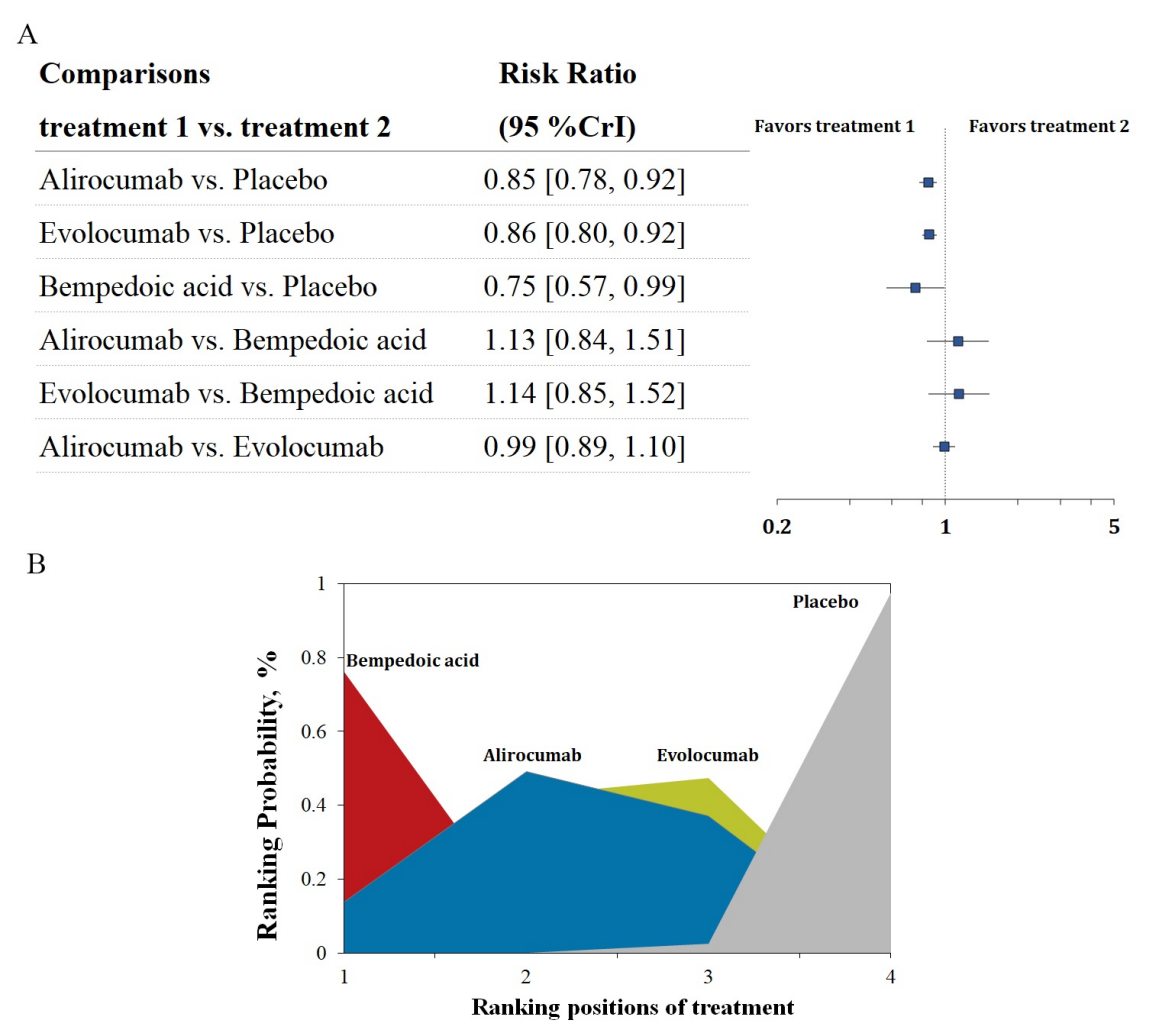
**Overview of the composite cardiovascular outcome.** (A) Summary RRs with 95% CrIs for the composite cardiovascular outcome. (B) Ranking plot showing the SUCRA values of each treatment agent: 86% for bempedoic acid, 59% for alirocumab, and 54% for evolocumab. SUCRA, surface under the cumulative ranking curve; RRs, risk ratios; CrIs, credible intervals.

**Fig. 5.**
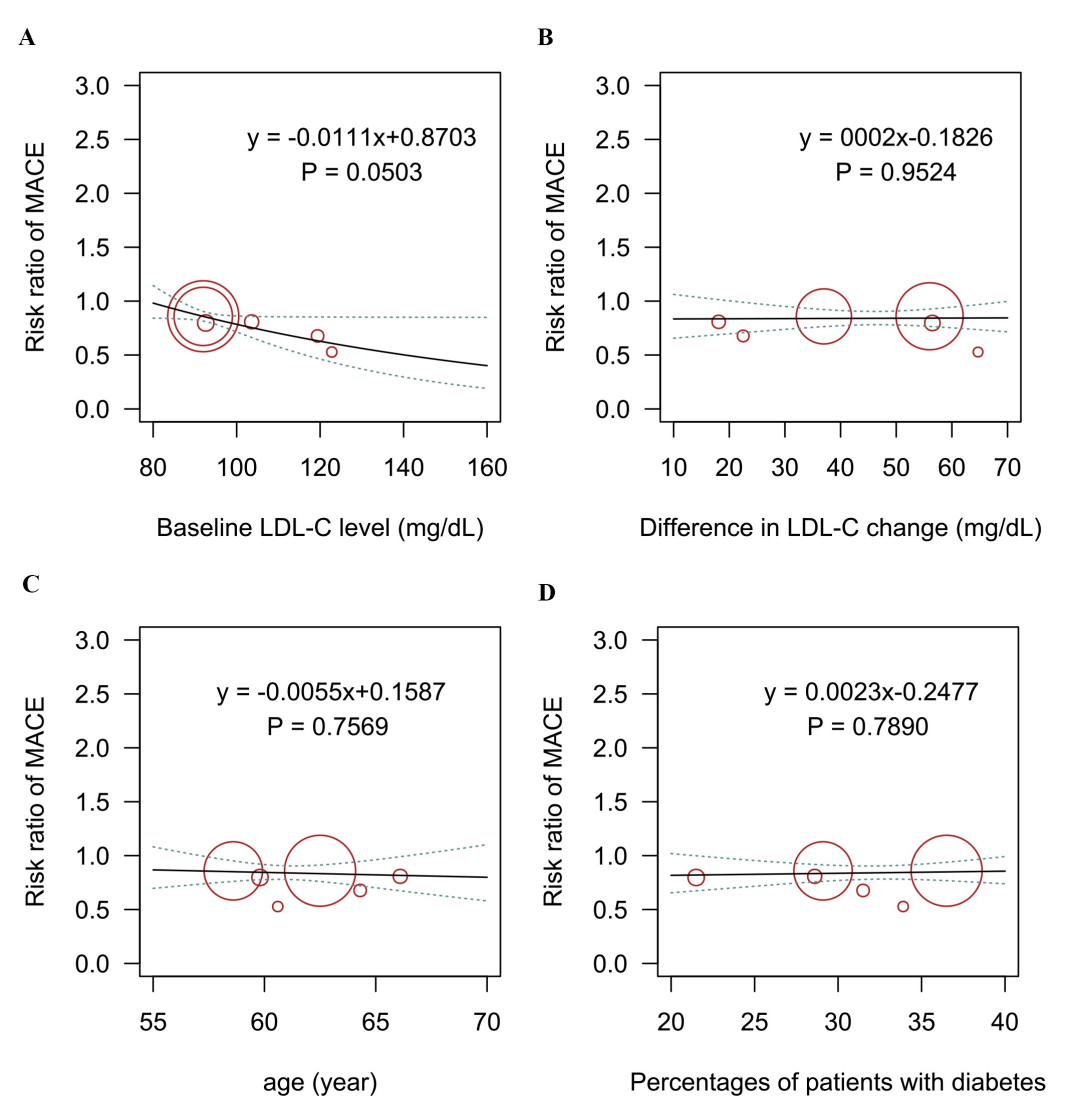
**Meta-regression analysis for the association of (A) baseline LDL-C level, (B) difference in LDL-C change, (C) age, (D) percentage of patients with diabetes on the incidence of the composite cardiovascular outcome.** LDL-C, low-density lipoprotein cholesterol; MACE, major adverse cardiovascular events.

Six trials with 52,802 patients reported data on the composite cardiovascular outcome. Three-node analysis (Table 2) showed both PCSK9 monoclonal antibodies (RR 0.85, 95% CrI 0.81–0.90) and bempedoic acid (RR 0.75, 95% CrI 0.57–0.99) reduced incidence of the composite cardiovascular outcome. The results of the four-node analysis are shown in Fig. 4A,B and **Supplementary Table 2**. Bempedoic acid was regarded as the best regimen that prevents major adverse cardiovascular events (RR 0.75, 95% CrI 0.57–0.99, SUCRA 0.86, compared with placebo), followed by alirocumab (RR 0.85, 95% CrI 0.78–0.92, SUCRA 0.59) and evolocumab (RR 0.86, 95% CrI 0.80–0.92, SUCRA 0.54). Meta-regression analyses were conducted to test the association between baseline LDL-C level, the difference in LDL-C change, and incidence of a composite outcome. The outcomes showed a statistically significant association between increased risks and baseline LDL-C level (*p* = 0.05; Fig. 5). Sensitivity analysis remained consistent by excluding the ODYSSEY LONG TERM trial (**Supplementary Table 3**).

**Fig. 6.**
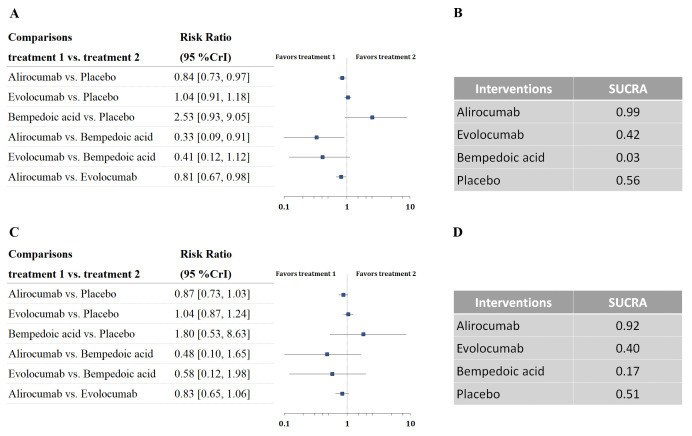
**Overview of other efficacy outcomes.** (A,B) Summary RRs with 95% CrIs and SUCRA values for all-cause death. (C,D) Summary RRs with 95% CrIs and SUCRA values for cardiovascular death. SUCRA, surface under the cumulative ranking curve; RRs, risk ratios; CrIs, credible intervals.

Seven trials with 53,101 patients reported data on all-cause mortality. The comparative effects of different LDL-C-lowering agents and all-cause mortality are shown in Fig. 6A,B. The analysis ranked alirocumab as the best treatment regimen to reduce all-cause death, with statistical significance (RR 0.84, 95% CrI 0.73–0.97, SUCRA 0.99). The analysis also revealed that alirocumab was superior to bempedoic acid (RR 0.33, 95% CrI 0.09–0.91) and evolocumab (RR 0.81, 95% CrI 0.67–0.98) in reducing the incidence of all-cause mortality.

Six trials with 52,802 patients reported data on cardiovascular mortality. The comparative effects of different LDL-C-lowering agents and all-cause mortality are shown in Fig. 6C,D. The analysis did not reveal statistical significance in any of the comparisons. Alirocumab was considered the best option to decrease cardiovascular mortality (RR 0.87, 95% CrI 0.73–1.03).

### 3.4 Risk of Bias and Quality of Evidence

**Supplementary Figs. 2,3** present risk-of-bias assessments. Four trials were regarded as having an overall low risk of bias, and two trials were rated as having an overall high risk of bias. The most notable risk of bias is the concern regarding incomplete outcome data. The main findings of the GRADE evaluation of certainty for primary outcomes are shown in **Supplementary Table 4**.

## 4. Discussion

### 4.1 Primary Findings

In this pooled analysis of seven trials comprising 53,106 patients, we combined direct and indirect results to establish updated evidence in order to investigate the preferred novel LDL-C-lowering therapeutics for the secondary prevention of cardiovascular events and new-onset diabetes. In the present analysis, several valuable observations were made. First, although all three treatment regimens were superior to placebo in decreasing the incidence of composite cardiovascular events, bempedoic acid ranked first in reducing the risk of a composite outcome. Second, bempedoic acid ranked first in reducing the incidence of new-onset diabetes. Furthermore, meta-regression analysis suggested that the elevated incidence of new-onset diabetes was positively related to a significant decrease in LDL-C levels. Third, alirocumab showed better efficacy in decreasing the incidence of all-cause mortality and cardiovascular mortality than the other treatment agents. Lastly, alirocumab was superior to other agents in decreasing SAEs.

Our analysis suggested that alirocumab ranked first in reducing all-cause mortality and cardiovascular mortality. This agent was superior to bempedoic acid and evolocumab in reducing all-cause mortality. The superiority of alirocumab in reducing mortality has been demonstrated in previous studies [25, 26]. The reason might be that alirocumab not only lowered LDL cholesterol to a greater extent, particularly in patients with high cardiovascular risk who were not at LDL-C target goals but also had a beneficial effect on improving plaque susceptibility [26, 27, 28].

### 4.2 Comparison with Other Research

Several meta-analyses have evaluated the efficacy of lipid-modifying treatments for the primary and secondary prevention of cardiovascular events. However, most of the previous studies were designed as direct meta-analyses, and none of them evaluated the relative effects of PCSK9 monoclonal antibodies and bempedoic acid on the secondary prevention of new-onset diabetes and cardiovascular events [25, 29, 30]. Only one network meta-analysis evaluated different types of non-statin therapies in patients with hypercholesterolemia [8]. This study selected change in LDL-C as the observed outcome. Evidence from this study indicated that alirocumab and evolocumab are anticipated to provide significant improvement in LDL-C levels in this group of patients.

This report is one of the largest systematic reviews in this field and the only network meta-analysis to date to investigate the relative safety and efficacy of novel LDL-C reduction therapies in the secondary prevention of new-onset diabetes and cardiovascular disease. The present study included a larger sample size than other studies, which led to more credible results, enhanced the accuracy of the estimation of treatment effects, and provided detailed suggestions regarding the choices of optimal treatment agents. In addition, this study used the SUCRA method to rank different treatment agents regarding various outcomes and assessed the quality of evidence of the new-onset diabetes outcome using the GRADE method. Moreover, meta-regression analyses were designed to examine the associations between different variables and the risk of new-onset diabetes, which will be useful for future research and information for clinicians.

The association between the degree of LDL-C reduction and new-onset diabetes (NOD) in patients treated with novel lipid-lowering treatment for secondary prevention of cardiovascular events remains to be elucidated. Recent study did not show LDL-cholesterol intensive lowering was associated with an increased incidence of NOD in an expanded population [31]. Our analysis observed that elevated incidence of new-onset diabetes might be related to a significant reduction in LDL-C level. The differences can be explained by these reasons. First, results from the meta-regression cannot infer any causality, but only association. Therefore, although we found an interaction between the difference in LDL-C changes and elevated NOD risk through meta-regression, more RCTs with large samples are still needed to validate the association between them. Second, the inclusion criteria were different. Our study focused on the secondary prevention of cardiovascular events in patients treated with novel lipid-lowering agents. Previous studies included trials of both primary prevention and secondary prevention of cardiovascular events and included patients treated with statin as well. Therefore, future research is needed to explore the connection between them.

### 4.3 Study Implications 

The US Food and Drug Administration has approved the administration of novel approaches including monoclonal antibodies targeting PCSK9 and bempedoic acid to reduce LDL-C levels. Moreover, recent studies have shown that PCSK9 inhibitors could exert cardiovascular event benefits in addition to LDL-C reduction by inhibiting the inflammatory response, autophagy and oxidative stress damage in endothelial cells [32]. However, it is not specified in the guidelines which type of LDL-C-lowering agent is the most suitable treatment method for this group of patients. Our analysis revealed that bempedoic acid might be superior to placebo and other agents in the secondary prevention of cardiovascular events and new-onset diabetes based on moderate-to-high quality evidence. However, it is suggested that the risk of new-onset diabetes might be associated with a reduction in LDL-C levels. Clinicians should take a considerate perspective of drug safety, effect, and cost effectiveness when choosing a specific agent.

Lipid-lowering therapy should receive particular attention in patients diagnosed with prediabetes and type 2 diabetes. It should be noted that more than 1/3 of diabetic patients have dyslipidemia. And the risk of atherosclerotic cardiovascular disease is four times higher in them compared to non-diabetic adults. Therefore, a more vigorous lipid management strategy is necessary for diabetic patients to maximize cardiovascular disease (CVD) prevention. Besides, the establishment of lipid-lowering therapy should be individualized, taking into account the patient’s baseline lipid level, LDL-C targets, renal function, etc. [33].

On the other hand, although recent studies have not shown that treatment with PCSK9 inhibitors increase the risk of NOD, there are safety worries about the PCSK9 targeting treatments, as loss-of-function variants of PCSK9 were associated with increased fasting plasma glucose levels [34]. Unlike PCSK9 inhibitors, bempedoic acid has the potential benefit of reducing the risk of NOD [35, 36, 37].

It should be noted that the latest guidelines from the American Diabetes Association (ADA) recommend the use of sodium-dependent glucose transporters 2 (SGLT 2) inhibitors for glycemic control and reduction of cardiovascular events in diabetic patients with co-morbid cardiovascular disease [38]. Specifically, in patients with acute myocardial infarction with diabetes undergoing percutaneous coronary intervention, SGLT 2 inhibitors treatment reduced in-stent restenosis-related events, and this beneficial effect was independent of glycemic control [39]. Moreover, the beneficial effect of SGLT 2 inhibitors on cardiovascular events in these patients was confirmed during a 39-month follow-up period [40]. Research has shown that in diabetic atherosclerotic plaques, SGLT 2 inhibitors was able to reduce plaque vulnerability by reducing plaque macrophage infiltration and matrix metallopeptidase (MMP) 9 expression, resulting in increased collagen content and plaque stability [41].

### 4.4 Study Limitations 

This analysis had several limitations. First, the dosage of the medication was not consistent across the included trials. Accordingly, the dose of PCSK9 inhibitors might influence the heterogeneity of the results. Second, differences exist regarding background lipid-lowering therapies. However, most studies used statins other than ezetimibe as background therapy. Third, this study was based on the study arm level but not the individual level, which restricted us from conducting a more detailed analysis. Fourth, most studies had a follow-up period of only 1 year. Longer periods of follow-up are required to make definitive conclusions regarding the comparative effects among different novel lipid-lowering agents. Fifth, there were some differences in baseline characteristics of patients among the included trials. For example, the proportion of patients with diabetes varied among the included trials. However, we performed the meta-regression analysis of these factors and the results did not show a significant impact on the primary outcome.

## 5. Conclusions

In conclusion, the present analysis showed that bempedoic acid ranked first in reducing the risk of a composite cardiovascular outcome. This agent also ranked first in reducing the risk of new-onset diabetes compared with placebo and evolocumab. Our analysis also suggests that the increased incidence of new-onset diabetes might be related to a significant reduction in LDL-C levels. Besides, alirocumab ranked first in decreasing all-cause mortality and cardiovascular mortality. Moreover, alirocumab was superior to bempedoic acid and evolocumab in decreasing all-cause mortality. Clinicians should be mindful of this issue when selecting the appropriate treatment agents. With the lack of available RCTs comparing all common treatment agents, this analysis provides novel and important evidence for clinicians to inform treatment decisions.

## Data Availability

All data generated or analyzed during this study are included in this published article and in its Additional file.

## Supplementary Material

Supplementary material associated with this article can be found, in the online version, at https://doi.org/10.31083/j.rcm2410286.
