# Supplementary material for: Novel Low-Density Lipoprotein Cholesterol Reduction Therapies for the Secondary Prevention of Cardiovascular Disease
Source: Rev Cardiovasc Med. 2023 Oct 8;24(10):286. doi: 10.31083/j.rcm2410286 (PMC11273145; doi:10.31083/j.rcm2410286)
Supplement: Supplementary file 1 [file 2153-8174-24-10-286-s1.zip › Supplementary Material.docx]

Supplemental e-material

| Table S1: Search Strategy  Table S2: Additional Statistical Methods in each outcome  Table S3: Sensitivity analysis by excluding the ODYSSEY LONG TERM trial  Table S4: GRADE summary for the primary outcomes  Figure S1: Study selection flowchart of randomized controlled trials |
| --- |
| Figure S2: Risk of bias summary |
| Figure S3: Risk of bias graph |

Table S1: Search Strategy

| **OVID Medline (adapted for other databases)** | |
| --- | --- |
| 1 | exp PCSK9 Inhibitors/ |
| 2 | (Alirocumab or SAR236553 or Evolocumab or AMG 145 or bempedoic acid or ETC-1002). ab,kw,ti |
| 3 | #1 or #2 |
| 4 | exp hypercholesterolemia/ or exp familial hypercholesterolemia/ |
| 5 | (Hyperlipidemia or Dyslipidemia).ab,kw,ti |
| 6 | #4 or #5 |
| 7 | #3 and #6 |
| 8 | exp randomized controlled trial/ |
| 9 | (random* or blind* or placebo or trial*).ab,kw,ti. |
| 10 | 8 or 9 |
| 11 | exp human/ |
| 12 | 10 and 11 |
| 13 | 7 and 12 |

Table S2. Additional Statistical Methods in each outcome

| **Outcomes** | **Global heterogeneity (*I^2^*)** | |
| --- | --- | --- |
|  | Pair-wise analysis | Network analysis |
| **Safety outcomes** |  |  |
| New DM | 0 | 0 |
| Serious adverse events | 0 | 0 |
| Neurocognitive disorders | 0 | 0 |
| **Efficacy outcomes** |  |  |
| The composite cardiovascular outcome | 15.49 | 15.48 |
| All-cause death | 0 | 0 |
| Cardiovascular death | 26.19 | 26.18 |

| **Intervention** | **RR (95% CrI) estimates derived from NMA** | | | | | | **SUCRA** | | | |
| --- | --- | --- | --- | --- | --- | --- | --- | --- | --- | --- |
|  | **Alirocumab vs. placebo** | **Evolocumab vs. placebo** | **Bempedoic acid vs. placebo** | **Alirocumab vs. bempedoic acid** | **Evolocumab vs. bempedoic acid** | **Alirocumab vs. Evolocumab** | **Alirocumab** | **Evolocumab** | **Bempedoic acid** | **Placebo** |
| **Safety outcomes** |  |  |  |  |  |  |  |  |  |  |
| Serious adverse events | **0.94 (0.89, 0.99)** | 1.00 (0.96, 1.04) | 1.06 (0.89, 1.26) | 0.89 (0.74, 1.06) | 0.95 (0.79, 1.13) | **0.94 (0.88, 1.00)** | 0.96 | 0.41 | 0.21 | 0.42 |
| New-onset diabetes | 0.95 (0.86, 1.05) | 1.05 (0.95, 1.16) | **0.72 (0.52, 0.99)** | 1.32 (0.93, 1.84) | **1.46 (1.03, 2.04)** | 0.91 (0.78, 1.05) | 0.60 | 0.09 | 0.97 |  |
| Neurocognitive disorders | 0.86 (0.68, 1.07) | 1.08 (0.90, 1.30) | 0.93 (0.40, 2.33) | 0.93 (0.36, 2.21) | 1.16 (0.46, 2.76) | 0.79 (0.59, 1.06) | 0.81 | 0.21 | 0.54 |  |
| **Efficacy outcomes** |  |  |  |  |  |  |  |  |  |  |
| MACE | **0.85 (0.79, 0.93)** | **0.86 (0.80, 0.92)** | **0.75 (0.57, 0.99)** | 1.14 (0.85, 1.53) | 1.14 (0.85, 1.52) | 1.00 (0.90, 1.12) | 0.57 | 0.56 | 0.86 | 0.01 |
| All-cause mortality | **0.85 (0.74, 0.99)** | 1.04 (0.91, 1.18) | 2.53 (0.94, 9.12) | **0.34 (0.09, 0.92)** | 0.41 (0.11, 1.12) | **0.82 (0.68, 0.99)** | 0.98 | 0.42 | 0.03 | 0.57 |
| Cardiovascular death | 0.89 (0.75, 1.05) | 1.04 (0.88, 1.24) | 1.79 (0.53, 8.59) | 0.50 (0.10, 1.70) | 0.58 (0.12, 2.00) | 0.85 (0.67, 1.08) | 0.89 | 0.40 | 0.18 | 0.53 |

Table 3. Sensitivity analysis by excluding the ODYSSEY LONG TERM trial.

RR: relative risk; CrI: credibility interval; SUCRA: surface under the cumulative ranking curve; NMA: network meta-analysis; MACE: major adverse cardiovascular events.

Table S4: GRADE summary for the primary outcomes

| **Intervention** | **New-onset diabetes** | **The composite cardiovascular outcome** |
| --- | --- | --- |
| **Compared with placebo** |  |  |
| Alirocumab | High | High |
| Evolocumab | High | High |
| Bempedoic acid | High | High |
| **Compared with bempedoic acid** |  |  |
| Alirocumab | Low^†§^ | Low^†§^ |
| Evolocumab | Low^†§^ | Low^†§^ |
| **Compared with evolocumab** |  |  |
| Alirocumab | Moderate^§^ | Moderate^§^ |

^§^downgraded one level for indirectness

^†^downgraded one level for imprecision


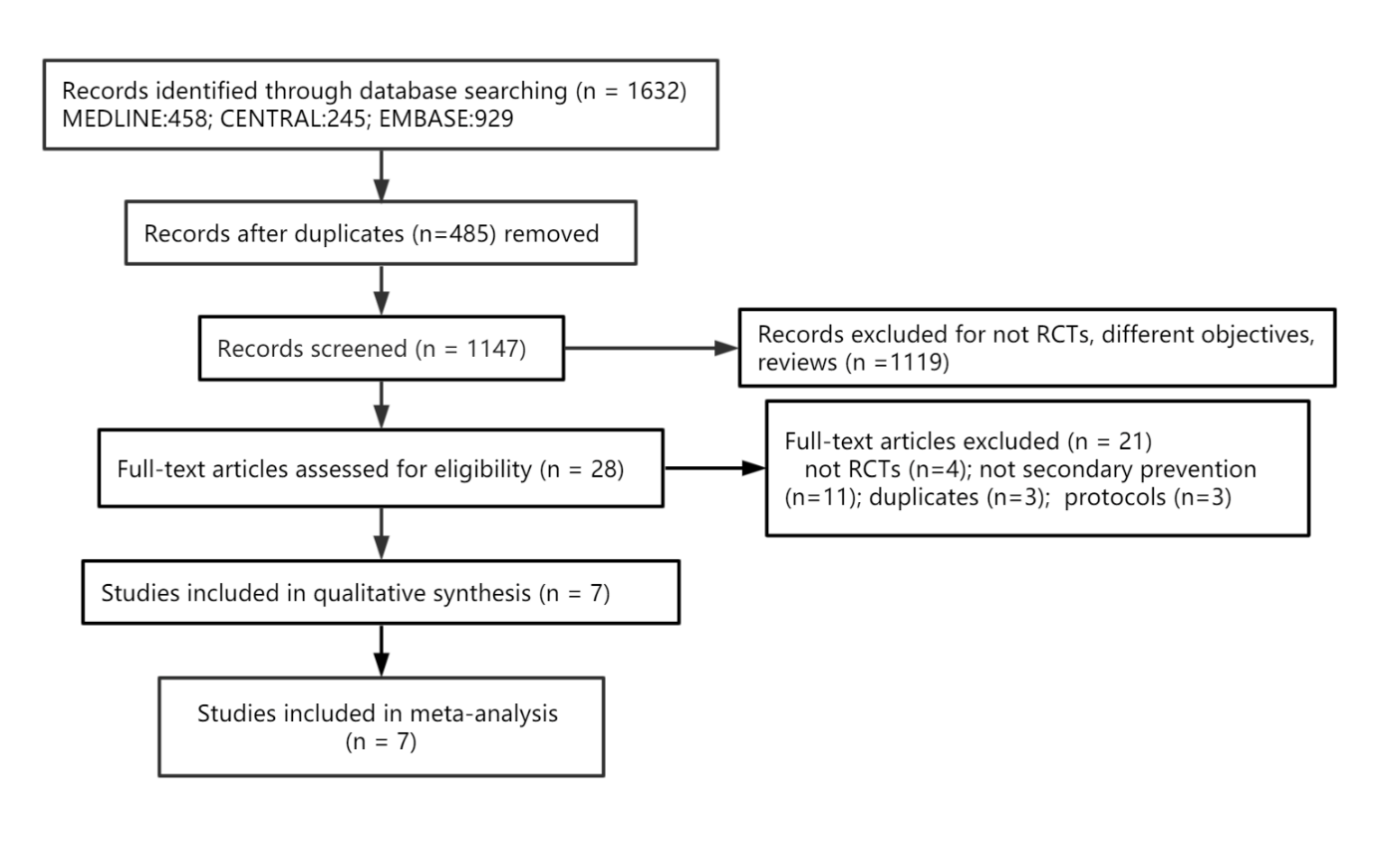


Figure S1: Study selection flowchart of randomized controlled trials


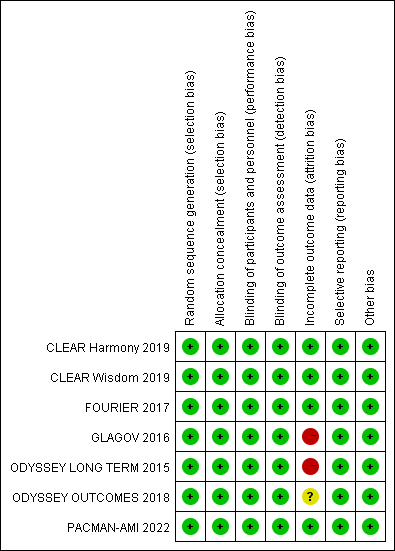


Figure S2: Risk of bias summary: review authors' judgements about each risk of bias item for each included study

**
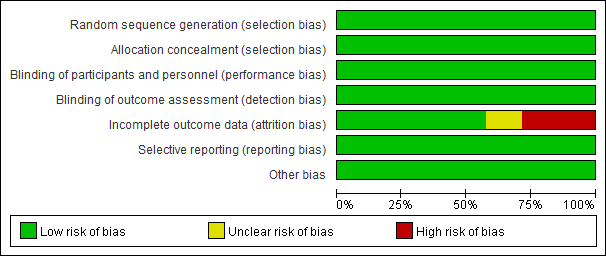
**

Figure S3: Risk of bias graph: review authors' judgements about each risk of bias item presented as percentages across all included studies
